# Recent advances in cardiac catheterization for congenital heart disease

**DOI:** 10.12688/f1000research.13021.1

**Published:** 2018-03-26

**Authors:** Sok-Leng Kang, Lee Benson

**Affiliations:** 1 Department of Pediatrics, Division of Cardiology, The Hospital for Sick Children, The Labatt Family Heart Center, The University of Toronto School of Medicine, Toronto, Canada; 2 Department of Pediatric Cardiology, Bristol Royal Hospital for Children, Bristol, BS2 OJJ, UK

**Keywords:** cardiac catheterization, heart, angiography

## Abstract

The field of pediatric and adult congenital cardiac catheterization has evolved rapidly in recent years. This review will focus on some of the newer endovascular technological and management strategies now being applied in the pediatric interventional laboratory. Emerging imaging techniques such as three-dimensional (3D) rotational angiography, multi-modal image fusion, 3D printing, and holographic imaging have the potential to enhance our understanding of complex congenital heart lesions for diagnostic or interventional purposes. While fluoroscopy and standard angiography remain procedural cornerstones, improved equipment design has allowed for effective radiation exposure reduction strategies. Innovations in device design and implantation techniques have enabled the application of percutaneous therapies in a wider range of patients, especially those with prohibitive surgical risk. For example, there is growing experience in transcatheter duct occlusion in symptomatic low-weight or premature infants and stent implantation into the right ventricular outflow tract or arterial duct in cyanotic neonates with duct-dependent pulmonary circulations. The application of percutaneous pulmonary valve implantation has been extended to a broader patient population with dysfunctional ‘native’ right ventricular outflow tracts and has spurred the development of novel techniques and devices to solve associated anatomic challenges. Finally, hybrid strategies, combining cardiosurgical and interventional approaches, have enhanced our capabilities to provide care for those with the most complex of lesions while optimizing efficacy and safety.

## Introduction

Over the last three decades, the pediatric cardiac catheterization laboratory has undergone a transformation from primarily a diagnostic tool to a modality for therapy. While this is an ongoing process, a variety of signature advances have taken place, altering management strategies. In the next few pages, we hope to highlight a few of those advances, which have improved the outcomes of children born with congenital lesions of the heart.

## Advances in imaging

### Three-dimensional rotational angiography and fusion imaging techniques

Three-dimensional rotational angiography (3DRA) is an emerging imaging modality that provides tableside, real-time acquisition of 3D volume rendered and cross-sectional images to aid the visualization of complex cardiac anatomy and navigation during diagnostic or interventional procedures ^1– 4^. Image acquisition is performed by rotation through an arc around the patient of the C-arm of the angiography system, equipped with flat-detector computer tomography (CT) ^1, 4^. The volume set is used to reconstruct the 3D structures of interest. These images can then be overlaid onto live fluoroscopy for road mapping during therapeutic procedures ^2, 4^. The so-registered 3D space can also be integrated with 3D datasets from magnetic resonance imaging (MRI) or CT studies.

**Figure 1.**
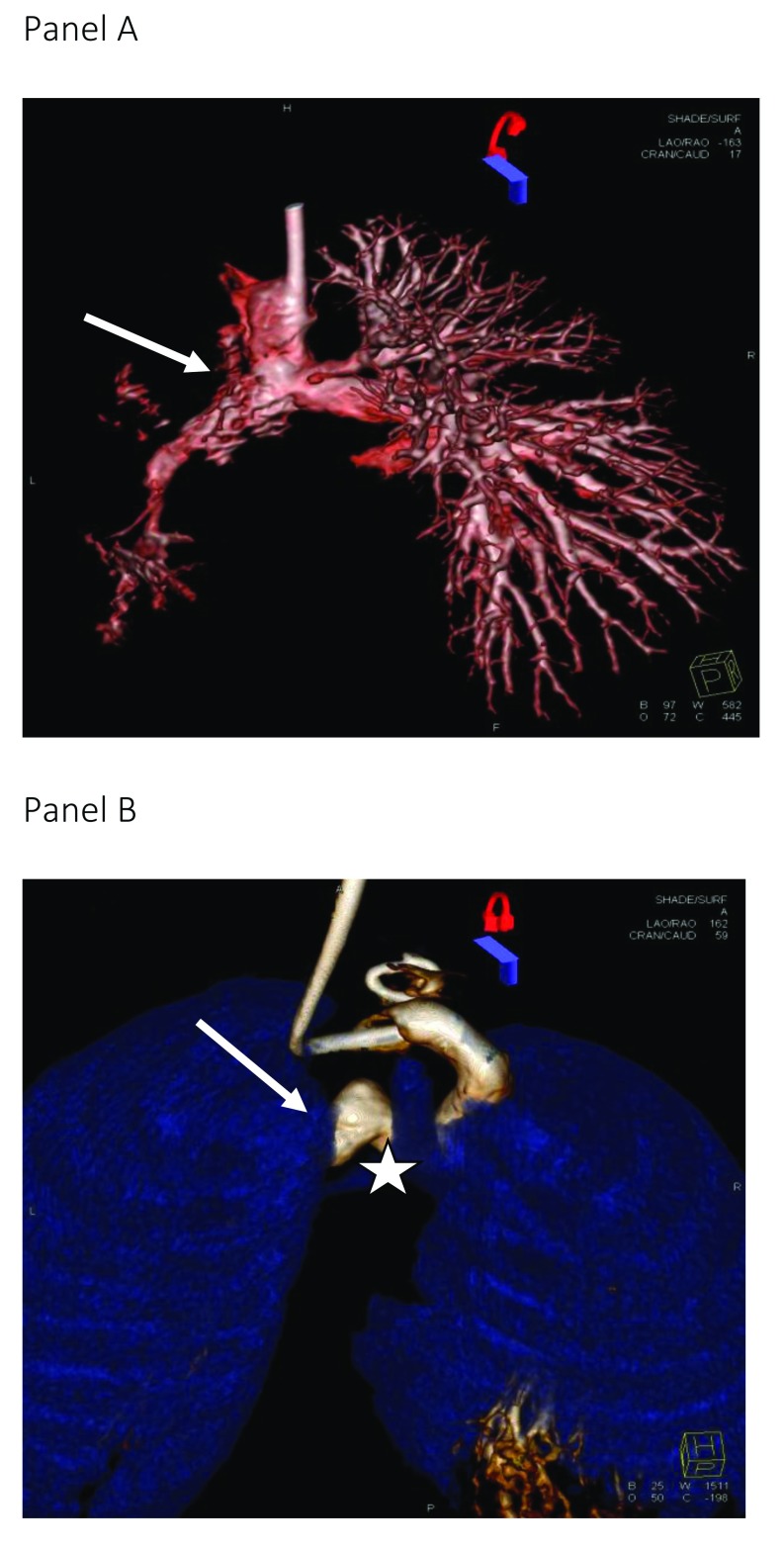
Rotational angiograms. Panel A: a three-dimensional (3D)-rotational angiogram in a child with severe left pulmonary artery stenosis related to the retained ductal stent (arrow). The injection was in the bidirectional cavopulmonary connection. Panel B: this reconstruction from a 3D-rotational angiogram shows the relationships between vascular structures; in this case, left bronchial stenosis (star) is due to vascular compression (arrow) following previous arch reconstruction.

Diagnostically, the advantage of 3DRA is its ability to profile the complexities of the cardiac anatomy from multiple projections, enhancing the appreciation of the spatial vascular relationships. To this end, several recent studies have reported the additive yield of using 3DRA compared to standard 2D biplane angiography ^2, 3, 5^. For example, in children with cavopulmonary connections, 3DRA can facilitate an understanding of the mechanisms underlying pulmonary artery (PA) stenosis and identify additional discrete proximal lesions at the anastomosis site ^5^. Additionally, the relationship of the trachea and bronchi to surrounding cardiovascular structures allows the assessment of airway anomalies and vascular compression frequently encountered in this population with significant implications for clinical management ( Figure 1) ^6, 7^.

In the interventional setting, 3DRA is useful for planning and guiding stenting for aortic coarctation and complex PA anatomies, percutaneous pulmonary valve implantation, interventions in the Fontan circulation, or after an atrial switch repair ^8– 12^. Image fusion from disparate modalities and image overlay during fluoroscopic procedures can provide continuous visualization of the target lesion in any angulation and guidance for catheters, wires, and device placement, enhancing procedural efficiency ^1^. As such, shortened procedural time reduces radiation exposure not only to the child but also to laboratory personnel and total contrast dose ^13, 14^. Current challenges, however, include the inability to gate for cardiac and respiratory motion and the potential for misalignment of multi-modality image registration and distortion of anatomy by rigid interventional equipment. Innovations to address these concerns, including non-rigid registration techniques to compensate for translational motion, and the “triple overlay” technique, allow co-registration of pre-procedural CT or MR angiography and intra-procedural 3DRA and transesophageal echocardiography with live fluoroscopy ^2, 11^.

### Three-dimensional printing, holography, and stereoscopic imaging for the interventional laboratory

**Figure 2.**
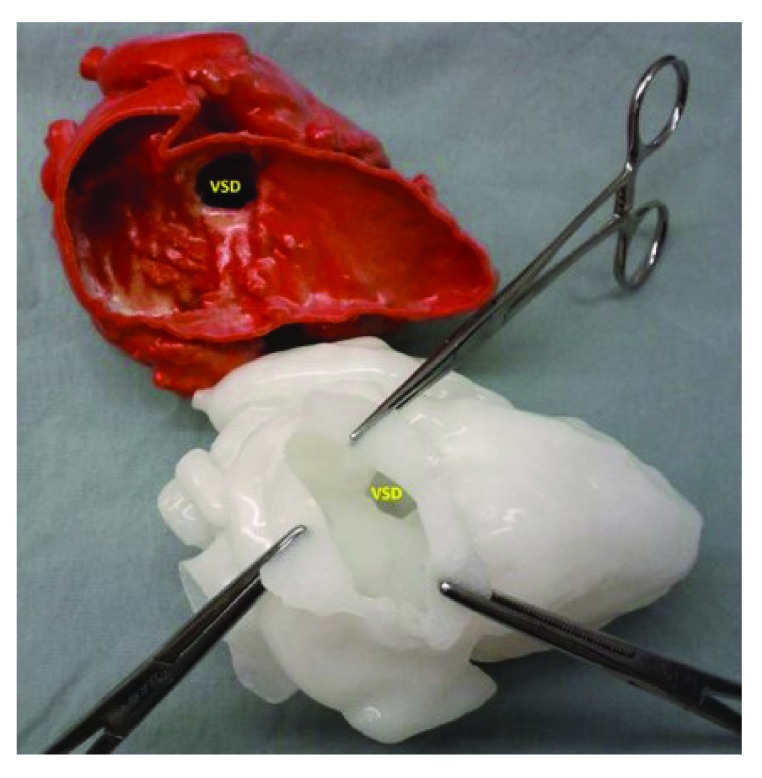
Three-dimensional modeling. The upper image is a three-dimensional model obtained from a magnetic resonance angiogram with the free wall of the right ventricle cut away revealing the location of the ventricular septal defect (VSD); the lower model, made from a soft pliable material, shows the appearance of the VSD as seen through a virtual incision in the right atrium, as would be seen by the surgeon.

Over the last two decades, catheter-directed interventions for congenital heart lesions have taken on a significant role in patient management. Indeed, many transcatheter interventions have become the standard of care for a number of abnormalities of heart valves, cardiac chambers, and proximal vessels ^15^. In this regard, children with complex congenital heart lesions, especially after complex operations, represent a challenge owing to their wide variation in complex morphology. One limiting factor in understanding and planning a percutaneous intervention is the limits of available 3D imaging modalities (MRI and CT) using images viewed on 2D screens. As such, these 2D representations make it inherently more difficult to appreciate the 3D relationships of cardiac structures unique to a particular intervention. Three-dimensional printing (also known as rapid prototyping, stereolithography, or additive manufacturing), while not a new technology, has only recently been used to fabricate physical models from 3D computerized imaging datasets ^16^. While the first medical applications were to produce surgical implants for oral and maxillofacial surgery and prosthetics for orthopedic surgery, the ability to generate a 3D model of complex cardiac anatomy has made this a tool for education, procedural planning, and device testing in both structural and congenital heart disease interventions ( Figure 2) ^17, 18^. The use of these constructed models representing true anatomical relationships has been a promising adjunct in planning a number of interventional procedures allowing for virtual device implantation ^19– 27^. However, expertise is required to generate these 3D models and the investment in resources is required to establish a 3D printing lab. As such, this technology has been limited thus far to teaching hospitals and research centers. In addition to the creation of physical models to view 3D anatomy, several augmented viewing modalities, using holography or stereoscopic imaging, are being evaluated.

## Radiation safety

Radiation safety awareness and techniques to reduce radiation exposure are essential for all procedures but have a special role in children with complex congenital cardiac lesions who often require long, and at times multiple, procedures during their lifetimes ^28^. A recent systematic review noted that radiation dose during pediatric cardiac catheterization remains varied and potentially substantial despite a downward trend in recent years ^29^. The observed decline in exposure estimates is attributed to improvement in physician awareness of dose optimization in tandem with technological advances ^29– 31^. However, the variation in radiation exposure amongst centers suggests that further initiatives towards minimizing radiation dose in children to “as low as reasonably achievable” (the ALARA principle) and standardization of practice are warranted ^29^. Comparisons between centers also highlight the inadequacy of fluoroscopy time alone as a metric of radiation dose estimate because of differences in programmed fluoroscopy modes, pulse rates, and cine acquisition frame rates ^32, 33^. Instead, recognition and comparison of actual radiation energy exposure such as dose area product and air kerma in relation to procedure types are more appropriate.

Several technical developments have impacted positively on the reduction of radiation exposure, notably the transition to flat panel detector (FPD) technology, which converts X-ray photon energy to digital signals more efficiently than image intensifiers ^30, 34– 36^. Innovation in FPD including increased pixel bit depth and usage of crystalline silicon rather than amorphous silicon combined with novel flat emitter X-ray tubes offers further reduction in radiation dose owing to better digitalization and lower detector noise ^37^. Additionally, alternative imaging techniques, such as transesophageal and/or intracardiac echocardiography or fusion of MRI/CT imaging with fluoroscopy, have been successfully used to reduce radiation exposure in various interventional procedures ^32, 33^.

## Specific applications

### Patent ductus arteriosus in preterm and low-birth-weight infants

A persistent patent ductus arteriosus (PDA) in the preterm newborn is often associated with important comorbidities (ventilator dependence, congestive heart failure, and failure to thrive) and increased mortality ^38, 39^. Definitive treatment strategies in this group remain debated, as both medical and surgical therapies have attendant risks ^40, 41^. Percutaneous ductal closure in these small newborns is generally limited by delivery sheath size, procedure-related hemodynamic instability, and the anchoring and retrievability characteristics of current devices ^42^. Although manufacturer recommendations for the most commonly used Amplatzer ductal occluders are a weight of 6 kg or more, there is no consensus on the minimum weight limit in practice.

Recently, there has been a growing body of evidence supporting the feasibility and efficacy of percutaneous PDA closure in the premature or small infant under 6 kg. The procedural success rates range from 88 to 94% in those with a median weight of between 2.5 and 6 kg ^43– 47^. Technical feasibility has also been demonstrated in extremely preterm (under 28 weeks) or very preterm (28 to under 32 weeks) infants under 2.5 kg, with the smallest noted in the literature weighing 755 g, having an uncomplicated closure with a 4 mm Amplatzer vascular plug II device ^44, 48– 52^. The adverse event rate was higher in the under 4 kg group, which was not unexpected given that premature infants are medically fragile ^45, 53– 55^, in keeping with the known inverse event rate related to patient weight at the time of catheterization ^56^. The increased risk of embolization is also noteworthy in low-weight and premature infants; hence, the retrievability of the occluder and duct morphology needs careful consideration ^46, 47^. There were no deaths attributable to the procedure reported in these studies.

Access-related complications, in particular acute arterial injury leading to vascular compromise of the extremity, is a significant concern when catheterizing these low-weight infants ^57, 58^. Some centers have avoided arterial access by adopting a transvenous-only approach guided by fluoroscopy and echocardiography ^33^. The risk of device-induced obstruction to the descending aorta or left PA (LPA) was low in these studies, and the majority tended to resolve over time with growth of the vessels ^43– 45, 52^. However, the severity of LPA stenosis may be underestimated because of outflow diversion to the right lung. Studies using scintigraphic perfusion imaging have shown evidence of decreased perfusion of the left lung after PDA occlusion, but the long-term clinical impact is unknown ^59, 60^. More recently, the introduction of a new microvascular plug (MVP™, Medtronic Inc.) for PDA closure in the extremely premature infant has shown promising results ^61, 62^. Advantages of the device include delivery through a microcatheter (<3 F), a flexible delivery cable, and diskless device design which minimizes the risk of device protrusion to the aorta or LPA.

### The role of right ventricular flow tract stenting in symptomatic neonates with Fallot’s tetralogy

**Figure 3.**
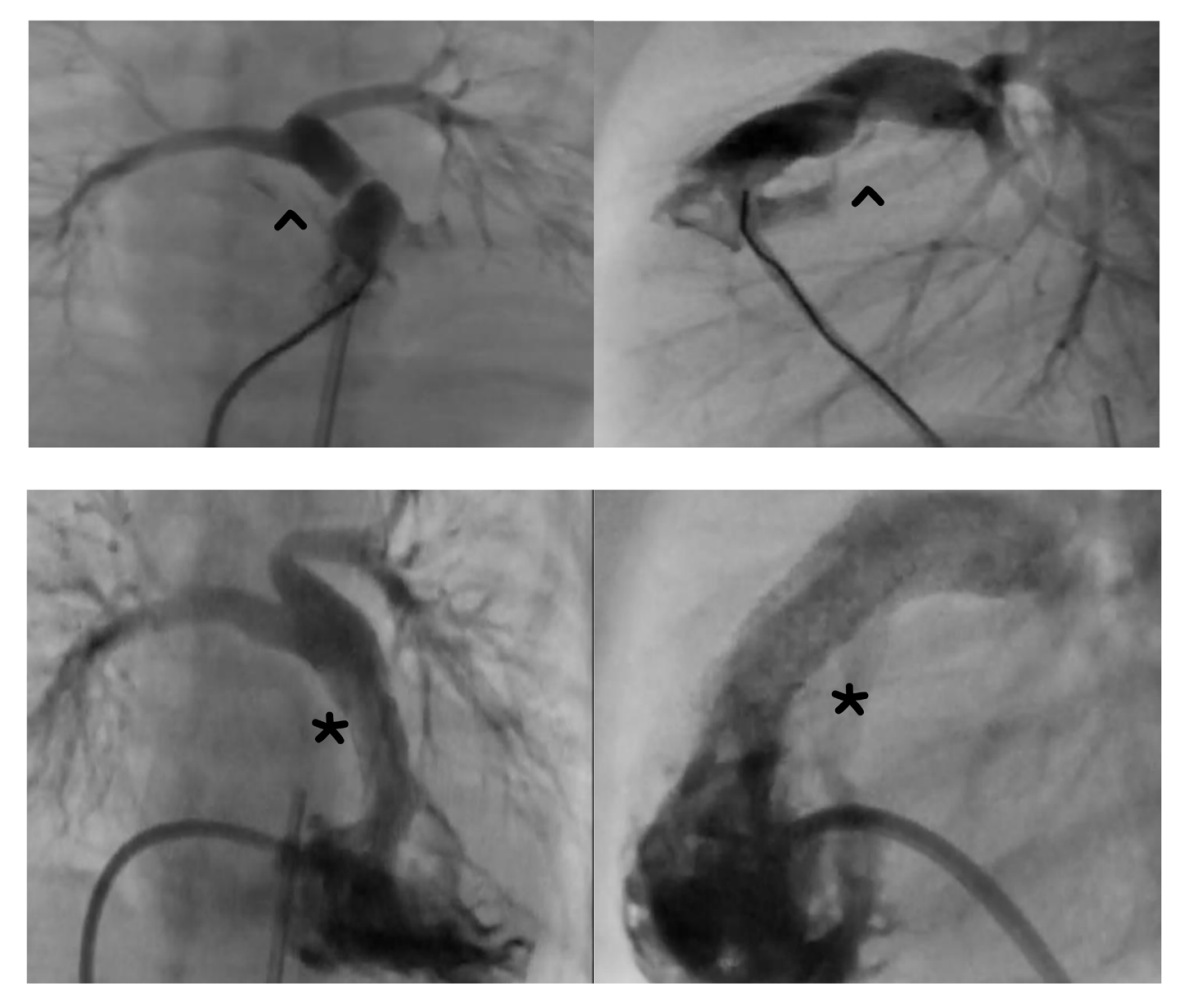
Hypoplastic pulmonary arteries and outflow stent. Upper panels from right ventricular angiograms show the obstructive right ventricular outflow tract (^) in a newborn with Fallot’s tetralogy. The lower panels show stent implantation (*) to enlarge the outflow and improve pulmonary blood flow, relieving the cyanosis.

Primary repair in infants with tetralogy of Fallot (TOF) with good-sized confluent central PAs is the standard of care with excellent outcomes. However, the ideal management strategy for the symptomatic (cyanotic) neonate with TOF and one or more adverse risk factors, such as prematurity, low birth weight, unfavorable PA anatomy, or pulmonary atresia or non-cardiac co-morbidities, remains debated ^63, 64^. Conventional palliation with the Blalock–Taussig (BT) shunt in this high-risk group can result in complications such as shunt stenosis or occlusion, distortion and differential growth of the PAs, and pulmonary overcirculation ^65– 67^. Balloon dilation of the pulmonary valve alone has inconsistent benefit owing to frequently associated muscular obstruction in the infundibulum. Stent implantation to enlarge the right ventricular outflow tract (RVOT) is now increasingly used as a bridging procedure to palliate the cyanosis and promote PA growth ( Figure 3).

Several small studies have documented favorable outcomes of RVOT stents in isolation since 2008, focusing on technical aspects and acute outcomes ^65, 67– 72^. More recently, Sandoval and colleagues provided comparative data on 180 infants with TOF who underwent RVOT stenting, early primary repair, or standard repair over 3 months of age ^63^. While the RVOT stented group had worse PA anatomy and clinical factors compared to the early intervention group, final clinical outcomes were comparable to infants who underwent standard repair. RVOT stenting resulted in relief of cyanosis and PA growth, allowing time for somatic growth and resolution of non-cardiac comorbidities until definitive repair ^63, 71^. Quandt and colleagues compared outcomes of RVOT stenting and BT shunts, reporting better PA growth and hence shorter duration of palliation before complete repair after stenting. The RVOT stent group also had a shorter intensive care unit (ICU) and hospital stay, whilst mortality until surgical repair was similar in both groups ^73, 74^.

Several disadvantages of RVOT stenting exist, notably the technical complexity and risk of inadvertent perforation of the RVOT in low-weight infants with membranous pulmonary valve (PV) atresia, who undergo radiofrequency perforation to open the RVOT prior to stenting ^63^. A hybrid perventricular route may offer a more controlled approach to PV perforation and RVOT stent placement while avoiding cardiopulmonary bypass and femoral vessel complications and has been reported in a few case series, including a neonate as small as 1.3 kg ^75, 76^. There is a high re-intervention rate for additional stent implantation (if the initial implant did not cover the full extent of the outflow tract) and PA dilation, although in this subset of infants the PAs are hypoplastic and require repeat procedures to encourage growth ^72^.

Previous retrospective comparisons of early primary repair versus a staged repair with a BT shunt in symptomatic neonatal TOF showed equivalent mortality and outcomes, although shunted patients had a greater likelihood of avoiding a transannular patch at the time of repair ^66^. However, there are currently no published data comparing the outcomes of early primary repair, BT shunt, and RVOT stent. Ultimately, the preferred palliative option, whether surgical or percutaneous, is likely to depend on local expertise, patient factors, and clinical condition at the time of intervention.

### The role of ductal stenting in the duct-dependent pulmonary circulation

**Figure 4.**
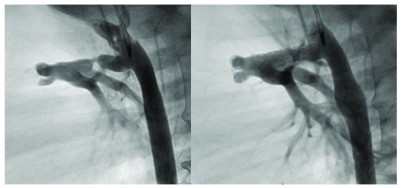
Ductal stent. The left panel details a stenotic arterial duct as it connects with the main pulmonary artery in a child with ductal-dependent pulmonary circulation. The right panel shows the appearance of the duct after the placement of a ductal stent to support the pulmonary blood flow.

While ductal stenting (DS) has been applied for well over two decades, the procedure has gained a wider acceptance as a palliative option for the cyanotic infant with a duct-dependent pulmonary circulation deemed unsuitable for primary repair ( Figure 4) ^77, 78^. The technique has been applied in various lesions, either leading to a biventricular repair or destined for univentricular palliation ^77, 79, 80^. Apart from avoidance of surgery and shunt-related adverse events, maintaining duct patency has been shown to promote significant and uniform PA growth compared to a BT shunt alone ^77, 81, 82^. The effective patency of the stented duct or PA growth potential was not significantly different between cases with a single source (ductal) of pulmonary blood flow or those with multiple sources of pulmonary blood flow ^77, 81^. In a few retrospective reports, DS has performed favorably and offered early survival advantage with improved hemodynamic stability compared to a BT shunt but with an increased likelihood of re-intervention prior to next-stage surgery ^78– 80, 83^.

One major disadvantage of DS is progressive neointimal proliferation and consequent endoluminal narrowing with an unpredictable timecourse ^82^. The adequacy of palliation provided by DS, however, depends on the clinical setting. When DS is performed after RVOT intervention for critical PS or PA, short-term patency is usually adequate. In other conditions, such as univentricular palliation prior to a Glenn procedure or biventricular repair of complex anatomy, a longer lifespan of the stented duct from 6 to 12 months is desirable to allow somatic growth ^82^. The impact of drug-eluting stents used in adult coronary artery disease has been investigated in this setting. Sirolimus-eluting stents implanted in the porcine arterial duct had higher patency rates compared with bare-metal stents with anti-proliferative action on ductal smooth muscle ^84^. Early trials of drug-eluting stents in neonates showed no clinically significant adverse outcomes; however, their clinical efficacy has yet to be evaluated ^85^.

### Percutaneous pulmonary valve implantation in the right ventricle to pulmonary artery conduit and native outflow tract

Percutaneous pulmonary valve implantation (PPVI) is a recognized alternative to surgical pulmonary valve replacement in selected patients with dysfunctional synthetic right ventricle-to-PA (RV-PA) conduits, bioprosthetic pulmonary valves, or homografts. Currently available percutaneous pulmonary valves include the Melody™ valve (Medtronic, Minneapolis, MN), which has a maximum outer diameter of 24 mm, and the Edwards Sapien™ valve system (Edwards Lifesciences Corp), with outer diameters of up to 29 mm.

Multiple studies have reported excellent procedural success and clinical efficacy of the Melody™ valve with thus far up to 7 years of follow up ^86– 89^. Published experience on the Sapien™ valve in the pulmonary position is comparatively less, although there are robust data supporting its use in the aortic position in high-risk or elderly patients with acquired aortic stenosis ^90, 91^. Early results of the COMPASSION (COngenital Multicenter Trial of Pulmonic VAlve Regurgitation Studying the SAPIEN Interventional THV) trial reported a good safety and efficacy profile, and several small studies have demonstrated comparable results to the Melody™ valve in the medium term ^92– 95^. With both the Melody™ and the Sapien™ valve, primary valve failure was rare and overall complication rates were low (0–5%) with reported mortality rates of up to 2% ^86– 89, 92– 95^. Coronary compression due to expansion of the implant occurred in less than 1% of cases and was significantly associated with an abnormal coronary artery course. Pre-implantation coronary artery compression testing is mandatory to avoid this potentially catastrophic complication ^51, 96^. Stent fractures which occurred with the Melody™ valve in the early experience have been addressed with routine RVOT pre-device implant stenting. The incidence of infective endocarditis after PPVI is estimated at approximately 3% per year with the Melody™ valve, whilst a lower incidence with the Sapien™ valve is suggested ^97^.

More recently, the application of both Melody™ and Sapien™ valves in the so-called “native” RVOT have been described in case reports and case series ^98– 101^. “Native” refers to the non-operated RVOT or one having a previous balloon pulmonary valvuloplasty/valvectomy for pulmonary stenosis or previous transannular patch repair for correction of TOF, which constitutes the vast majority of patients with a dysfunctional RVOT. The potentially distensible tissue in the contractile “native’” RVOT and the absence of a pre-existing “scaffold” such as in a conduit or bioprosthetic valve presents potential problems with implant valve stability, a paravalvular leak, or framework fracture. To overcome such issues, several techniques of pre-stenting the RVOT have been developed to create a “landing zone” for the selected transcatheter valve ^11^. Implantation of the Melody™ valve in the branch PAs has also been described ^102^.

**Figure 5.**
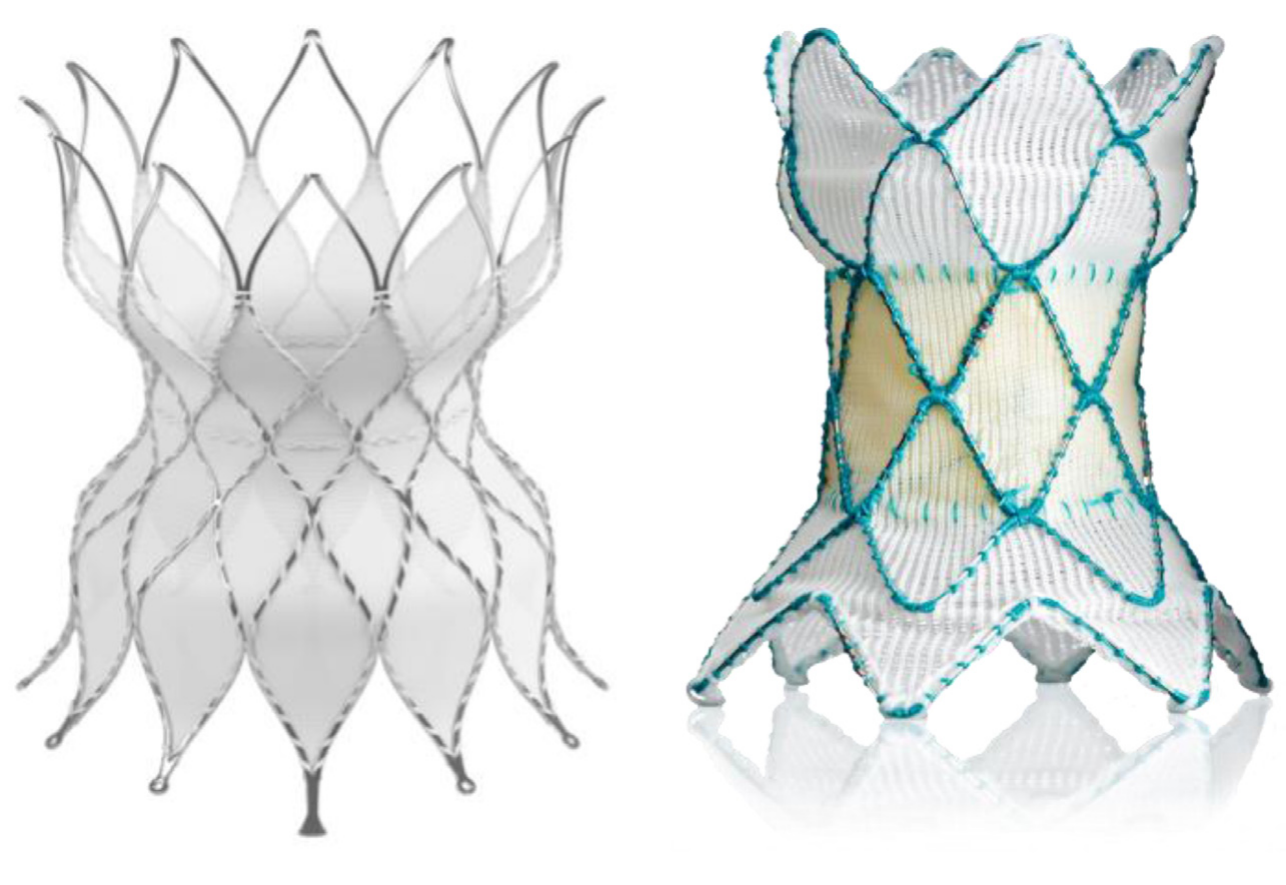
Percutaneous pulmonary valves for the large outflow tract. The left panel is a picture of the Edwards Alterra™ stent, designed to be placed in the right ventricular outflow tract in patients after transannular patch repair for Fallot’s tetralogy. The nitinol stent hosing (seen in the photo) provides a landing zone for a 29 mm Sapien™ valve. The right panel is a photo of Medtronic’s Harmony™ valve. This valve stent design has porcine pericardial valve leaflets sewn into the nitinol framework.

For the dilated “native” RVOT often encountered after a transannular patch repair for TOF, hybrid approaches of RVOT plication through a limited sternotomy or thoracotomy and subsequent percutaneous or perventricular delivery of the Melody™ valve have been explored ^103– 105^. Paralleling the limited application of these first-generation PPVIs in the “native” RVOT is the development of self-expanding percutaneous valves such as the Harmony™ valve (Medtronic Inc, Minneapolis, USA), the Venus P™ valve (Medtech, Shenzhen, China), and a rendezvous Alterra™ stent (Edwards Lifesciences) as a landing zone for a Sapien™ valve ^106– 111^. The flared ends of these systems are designed to provide stability in the large pulsatile outflows, whilst the self-expanding nitinol frames adapt to various outflow anatomies ( Figure 5). Other concepts in development (in animal studies) include implantation of expandable conduits in the RVOT that can be dilated sequentially with growth or replaced with a percutaneous valve ^112– 114^.

### Transcatheter tricuspid and mitral valve replacement

The rapidly expanding transcatheter heart valve technology has been applied to the treatment of atrioventricular valve dysfunction with a growing experience in the tricuspid valve position. Implantation of percutaneous valves into degenerated bioprostheses (valve-in-valve, VIV) or into annuloplasty rings (valve-in-ring, VIR) is an attractive alternative to surgery in the high-risk and often-debilitated patient with acquired or congenital cardiac disease ^115– 118^. A large multicenter experience of tricuspid VIV implantation has recently been published, using both the Melody™ and the Sapien™ valves with favorable outcomes at more than a year follow up ^117^. The majority of patients were relatively young (median age 40 years), and over half had tricuspid valve disease associated with congenital heart disease including Ebstein’s anomaly, intrinsic tricuspid valve (TV) abnormalities, or TV injury related to previous surgery or catheter intervention. There was an excellent procedural success of 99%, with significant improvements in the degree of regurgitation or stenosis and symptomatic improvement. The 30-day mortality was 3.3%, and estimated freedom from tricuspid re-intervention at 1 year was 83%. The extension of the tricuspid VIV concept to VIR is considerably more challenging owing to larger diameters and geometric variability of surgically placed rings. Although this approach is technically feasible and clinically effective in reducing tricuspid regurgitation, paravalvular regurgitation has been common ^119, 120^.

Transcatheter mitral valve replacement remains in its early clinical stages ^121^. Mitral VIV implantation is more difficult to accomplish owing to difficulty in coaxial alignment of the percutaneous valve within the existing valve. The majority of implantations are performed using a direct transapical approach with the larger diameter Sapien™ valves ^122– 124^. A transvenous, transseptal technique utilizing an apical rail to facilitate the delivery of Melody™ valve into the dysfunctional mitral prosthesis has also been described ^118^. To date, the results of mitral VIV implantations within a high-pressure hemodynamic environment have shown good valve performance with low transvalvular gradients and low rates of paravalvular regurgitation in the short term ^122– 125^. Applying the Sapien™ valve to surgically implanted ring (VIR) is even less appealing because of incomplete sealing of the variable and D-shaped annuloplasty rings and the risk of causing left ventricular outflow tract obstruction. Recent successful case reports of the Melody™ VIR technique, however, offer significant promise ^126, 127^. Compared to Sapien™ valves, the Melody™ valve has a longer stent that is covered throughout its length, which provides good sealing at the subvalvar level ^128^. Transcatheter valve-in-native-ring for calcified native mitral stenosis has also been reported in high-risk adult patients ^129– 131^.

In infants and children with severe mitral stenosis or regurgitation associated with congenital heart disease, the Melody™ valve as a surgical implant has shown promising results after failed attempts at primary mitral valve repair ^132, 133^. The Melody™ valve has the potential for future expansions with child growth and is a viable option in the lack of appropriately sized mitral valve prostheses in these small children; however, further study is required to determine longer-term durability and safety.

## Conclusion

Recent years have seen the rapid development of imaging and device technologies as well as percutaneous interventions in a variety of congenital cardiac lesions, with an increased application of percutaneous therapies to a broad range of patients. It is important, however, to remember that long-term outcomes for many such novel interventions are lacking, and rigorous prospective studies and data surveillance are required to determine safety and efficacy profiles before these become standard of care. Future innovations and growing experience in this field, in addition to increased collaboration between surgeons and interventionists, will undoubtedly continue to expand transcatheter options in the management of congenital heart disease, further improving the quality of life for the child and adult with congenital heart disease. This short review touches on some of the highlights that have been developed over the last decade. A number of percutaneous procedures (not mentioned) have become standard of care in many centers, and with continued diligence we can anticipate the continued application of such therapies.
